# The effects of elective abdominal surgery on protein turnover: A meta-analysis of stable isotope techniques to investigate postoperative catabolism

**DOI:** 10.1016/j.clnu.2022.01.023

**Published:** 2022-03

**Authors:** Matthew Jaconelli, Paul L. Greenhaff, Philip J. Atherton, Dileep N. Lobo, Matthew S. Brook

**Affiliations:** a MRC-Versus Arthritis Centre for Musculoskeletal Ageing Research, Metabolic and Molecular Physiology, University of Nottingham, Queen's Medical Centre, Nottingham, UK; b School of Life Sciences, University of Nottingham, Queen's Medical Centre, Nottingham, UK; c National Institute for Health Research (NIHR) Nottingham Biomedical Research Centre, Nottingham University Hospitals and University of Nottingham, Queen's Medical Centre, Nottingham, UK; d School of Medicine, University of Nottingham, Royal Derby Hospital, Derby, UK; e Gastrointestinal Surgery, Nottingham Digestive Diseases Centre, Nottingham University Hospitals and University of Nottingham, Queen's Medical Centre, Nottingham, UK

**Keywords:** Surgery, Postoperative, Muscle protein synthesis, Muscle protein breakdown, Stable isotope studies, Meta-analysis, AV, Arterio-venous, CI, Confidence interval, DI, Direct-incorporation, EP, End-product, FSR, Fractional synthetic rate, GC-IRMS, Gas chromatography-isotope ratio mass spectrometry, GI, Gastrointestinal, IQR, Interquartile range, RCT, Randomized controlled trial, SD, Standard deviation, SMD, Standardized mean difference

## Abstract

**Background & aims:**

Elective surgery induces skeletal muscle wasting driven by an imbalance between muscle protein synthesis and breakdown. From examination of diverse stable isotope tracer techniques, the dynamic processes driving this imbalance are unclear. This meta-analysis aimed to elucidate the mechanistic driver(s) of postoperative protein catabolism through stable isotope assessment of protein turnover before and after abdominal surgery.

**Methods:**

Meta-analysis was performed of randomized controlled trials and cohort studies in patients undergoing elective abdominal surgery that contained measurements of whole-body or skeletal muscle protein turnover using stable isotope tracer methodologies pre- and postoperatively. Postoperative changes in protein synthesis and breakdown were assessed through subgroup analysis of tracer methodology and perioperative care.

**Results:**

Surgery elicited no overall change in protein synthesis [standardized mean difference (SMD) −0.47, 95% confidence interval (CI): −1.32, 0.39, p = 0.25]. However, subgroup analysis revealed significant suppressions via direct-incorporation methodology [SMD -1.53, 95%CI: −2.89, −0.17, p = 0.03] within skeletal muscle. Changes of this nature were not present among arterio-venous [SMD 0.61, 95%CI: −1.48, 2.70, p = 0.58] or end-product [SMD -0.09, 95%CI: −0.81, 0.64, p = 0.82] whole-body measures. Surgery resulted in no overall change in protein breakdown [SMD 0.63, 95%CI: −0.06, 1.32, p = 0.07]. Yet, separation by tracer methodology illustrated significant increases in urinary end-products (urea/ammonia) [SMD 0.70, 95%CI: 0.38, 1.02, p < 0.001] that were not present among arterio-venous measures [SMD 0.67, 95%CI: −1.05, 2.38, p = 0.45].

**Conclusions:**

Elective abdominal surgery elicits suppressions in skeletal muscle protein synthesis that are not reflected on a whole-body level. Lack of uniform changes across whole-body tracer techniques are likely due to contribution from tissues other than skeletal muscle.

## 1 Introduction

Skeletal muscle wasting is a key feature of the metabolic response to surgery, known to complicate postoperative recovery and impair clinical outcomes [1]. Although this phenomenon has been observed since early investigations into the metabolic perturbations that occur as a result of trauma and surgery [[2], [3], [4]], the underlying dynamic drivers of these metabolic changes within muscle are yet to be fully defined. Loss of skeletal muscle mass must occur through a chronic imbalance between muscle protein synthesis and muscle protein breakdown, with stable isotope techniques that calculate fractional synthetic rate currently considered a ‘gold standard’ for the measurement of muscle protein synthesis [5]. These techniques have been employed in the perioperative setting [[6], [7], [8]] and have shown distinct synthetic responses when compared with other stable isotope tracer techniques that quantify arterio-venous protein kinetics within the blood [9,10] or tracer kinetics within urinary end-products [11,12]. Comparisons of protein breakdown rates across tracer methodologies are limited by challenges in the assessment of skeletal muscle protein breakdown due to both underlying assumptions in kinetic modelling [13,14] and protocols ill-suited to clinical populations [14,15]. Hence, there is a paucity of information on fractional breakdown rates in the surgical patient, with stable isotope measures of protein breakdown predominantly reflecting whole-body kinetics. Taken together, the dynamic changes driving postoperative muscle wasting are unclear.

Major abdominal surgery has been shown to elicit systemic metabolic dysregulation within skeletal muscle, including alterations in catabolic and inflammatory signaling pathways [16]. In addition, traditional surgical care for these patients has often prescribed prolonged periods of preoperative fasting [17], putting these patients at great risk of postoperative skeletal muscle wasting through energy deficits [18]. Even in light of enhanced recovery programs aimed at reducing the metabolic stress response to surgery [19], in part through recommendations on the avoidance of preoperative fasting and early resumption of oral nutrition postoperatively [1], a recent audit of UK hospitals has illustrated elective surgical procedures - constituted by approximately 70% upper GI, colorectal or general surgery - to routinely involve preoperative fasting of >12 h for food (73% incidence) and clear fluids (21% incidence) [20]. A synthesis of stable isotope studies quantifying protein kinetics in the patient undergoing abdominal surgery may elucidate the changes in protein turnover driving postoperative catabolism, while informing future care strategies aimed at minimizing skeletal muscle wasting to improve patient outcomes and recovery.

The aims of this meta-analysis were to:

• determine postoperative changes in protein kinetics driving skeletal muscle catabolism, through a synthesis of studies utilizing stable isotope research methodologies across a range of elective abdominal surgical procedures and clinical care.
• assess the impact of perioperative care strategies such as nutritional support, neuraxial blockade and minimally invasive (laparoscopic) surgical approaches, and
• evaluate the postoperative time-course of protein turnover responses.

## 2 Methods

### 2.1 Search strategy

Electronic searches were performed in PubMed, MEDLINE and Cochrane Library databases to identify suitable articles (i.e. evaluating either whole-body or skeletal muscle protein turnover using stable isotope tracer methodology in adult patients undergoing elective abdominal surgery) published between 01 January 1990 and 08 November 2020. This date restriction was imposed due to the validation of several clinically suitable stable isotope techniques for protein metabolism occurring throughout the 1980s [[21], [22], [23], [24]]; studies which contributed to increased interest into the effects of surgical trauma on protein turnover during the late 1980s [25,26] and to the development of commercially available gas chromatography-isotope ratio mass spectrometers (GC-IRMS) capable of capturing increased signal sensitivity within complex biological matrices [27]. The search terms [“surgery”] AND [“muscle” OR “protein”] AND [“stable isotope” OR “tracer” OR “turnover”] were used to search each database by title and abstract. The bibliographies of all studies which fulfilled the inclusion criteria were manually reviewed to aid in locating additional eligible articles. There were no language restrictions in place during article selection. This meta-analysis was conducted in accordance with the guidance of the PRISMA statement [28] and conforms to AMSTAR-2 guidelines [29].

### 2.2 Study selection

Articles were screened for suitability by title and abstract on two separate occasions by one reviewer (MJ) and verified by a senior reviewer (MSB). Articles were deemed eligible if they described at least one adult patient cohort undergoing elective abdominal surgery, with pre- and postoperative measures of whole-body or skeletal muscle protein turnover through stable isotope tracer methodologies. Postoperative measures were included if they were performed within two weeks of surgery. Patients receiving a variety of nutritional and analgesic regimens were included due to the inherent heterogeneity of surgical care across different hospital settings and procedures. However, any patient cohort that was specified by study authors to be undergoing non-conventional perioperative care or receiving non-standard drug administration or hormone therapy was excluded. Pre- and postoperative measures of protein turnover had to be performed during the same nutritional state for each patient group, specifically; pre- and postoperative measures had to be both in the postabsorptive or postprandial state to enable accurate comparisons of protein turnover within patients, due to the dynamic regulation of muscle protein turnover with feeding [30]. Patients undergoing emergency, transplant or reconstructive procedures or suffering from burns, preoperative trauma, metabolic disorders, prolonged anti-inflammatory or antibiotic medication, organ dysfunction or failure were excluded. Abdominal surgery was defined as general, urological, or gynecologic, with vascular procedures omitted. Only studies on patients undergoing abdominal surgery were included in this analysis to improve homogeneity in postoperative protein turnover responses, as there is evidence to suggest that the catabolic response to surgery is relative to the magnitude of trauma [11,12,31]. Further, ischemia and reperfusion effects have been shown to impact protein turnover rates within an animal model [32], with great variation in postoperative protein turnover responses in humans previously being demonstrated within a heterogenous abdominal surgical cohort containing vascular procedures [33]. Patients were deemed adults if they were 18 years or older, with all pediatric studies being ineligible. Records containing duplication of study results were omitted, with only the primary publication taken forward for inclusion. Duplication of articles eligible for screening were assessed by title using Python programming language (version 3.6.5), with a subsequent manual check to ensure the full removal of duplicate articles. Duplication of study results was checked manually during full-text screening of eligible articles. For any article where fulfilment of the inclusion criteria was unclear, inclusion was discussed by two reviewers (MJ and MSB) and a final decision was made.

### 2.3 Data extraction

Data were extracted by one author (MJ) on two separate occasions and cross-compared to ensure accurate inclusion of article information. These data were then reviewed by a second author (MSB). Where studies contained more than one patient cohort, these cohorts were combined to prevent unit-of-analysis-error in accordance with recommendations from the Cochrane Handbook for Systematic Reviews of Interventions [34]. Data were additionally collected on patient demographics, surgical preparation and underlying conditions necessitating surgical intervention. Where studies did not contain the necessary information, study authors were contacted for retrieval. Where studies did not report the mean and standard deviation of protein turnover measures; median and interquartile ranges were converted to means and standard deviations according to the technique described by Hozo et al. [35]. This technique takes the median as the best estimate of the mean and calculates the SD as follows:

$$SD=\frac{Upper\;Limit\;of\;IQR-Lower\;Limit\;of\;IQR}{1.35}$$

Where relevant, risk of bias for randomized controlled trials (RCTs) was assessed using the Cochrane Collaboration Tool [36]. Publication bias was assessed via funnel plots and tested for via Pustejovsky's and Rodgers' [37] modified test of linear regression for standardized mean difference effect sizes.

### 2.4 Outcome measures

The primary outcome was to detect changes before and after surgery in whole-body or skeletal muscle protein turnover measured via stable isotope tracer methodology. Secondary outcomes aimed to investigate the influence of tracer methodology, severity of trauma (laparoscopic *vs*. open procedures), nutritional support and anesthetic regimen on the primary outcome measures. Meta-analysis of these outcomes was achieved through subgroup analyses. The population, intervention, comparator group and outcome (PICO) are summarized in Supplementary Table 1.

### 2.5 Statistical analyses

Data were prepared in Excel spreadsheet format and imported into R programming language (version 4.1.0, The R Foundation for Statistical Computing, http://www.R-project.org). The ‘meta’ package was used for data analysis. Continuous variables are quoted as standardized mean difference (SMD) with 95% CI and were analyzed using a random-effects, inverse-variance model. The DerSimonian-Laird estimator [38] was used to calculate heterogeneity variance, τ2, with Knapp-Hartung adjustments [39] applied in the calculation of confidence intervals around pooled study effects. Forest plots were generated, with statistical significance determined as p < 0.05 with 2-tailed testing. Study heterogeneity was assessed by *I*^2^ statistic [40], with <25% representing low heterogeneity, 25–50% representing moderate heterogeneity and >50% representing high heterogeneity. Meta-regression was performed to investigate time as a continuous variable across postoperative sampling timepoints, to determine whether this impacted the assessment of postoperative protein turnover.

### 2.6 Protocol registration

The protocol for this meta-analysis was registered on the Prospero database (www.crd.york.ac.uk/prospero), registration number: CRD42021178987.

## 3 Results

**Fig. 1:**
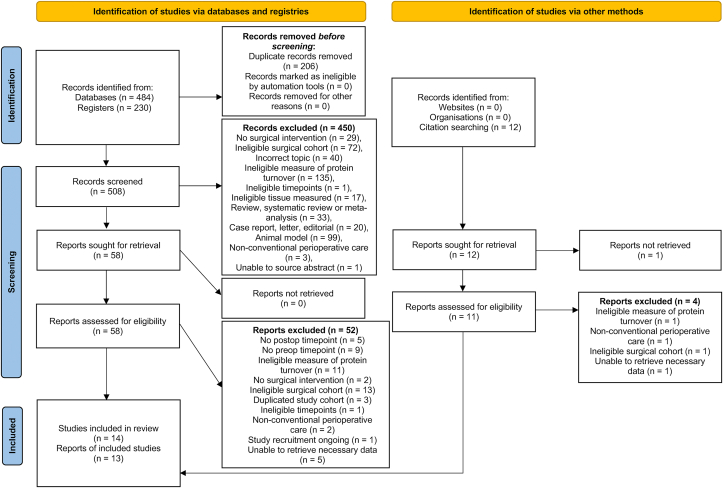
PRISMA flow-diagram detailing article identification for meta-analysis.

From the 714 studies identified through electronic database searches, 14 studies [[6], [7], [8], [9],^11^,^12^,^31^,[41], [42], [43], [44], [45], [46], [47]] reporting on 190 patients, were included (Fig. 1). Of these, twelve [[6], [7], [8], [9],^11^,^12^,[41], [42], [43], [44], [45], [46]] reported measures of protein synthesis (154 patients) and nine [9,11,12,31,[41], [42], [43],^46^,^47^] reported measures of protein breakdown (139 patients). From the studies reporting more than one postoperative timepoint [9,11,12,31,42,43], the timepoint closest to surgery was used for analyses, and where differential feeding was involved, its corresponding preoperative baseline value. The full-text from one eligible study [45] was unable to be sourced and attempts to contact the corresponding authors were unsuccessful. However, the abstract contained the necessary information required for inclusion and as such the decision was made between reviewers (MJ and MSB) to include data from this article in the meta-analysis. There were six studies [10,[48], [49], [50], [51], [52]] that fulfilled inclusion criteria but did not contain the necessary information needed for synthesis in the meta-analysis, with the authors being unable to provide the necessary information upon request. These studies were subsequently omitted from the analyses (Supplementary Table 2).

### 3.1 Risk of bias

**Fig. 2:**
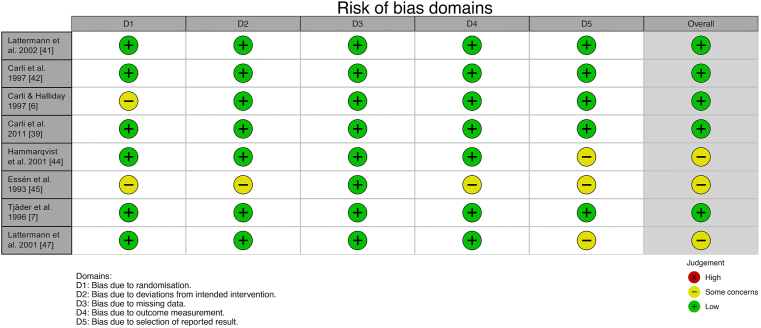
Risk of bias of the included randomized controlled trials.

Of the 14 studies included in this meta-analysis, eight were RCTs (predominantly investigating parameters related to perioperative catabolism) [6,7,[41], [42], [43], [44], [45],^47^] and six were cohort studies [8,9,11,12,31,46]. However, none of the RCTs involved randomization of the respective variables of interest within the subgroup analyses performed, with randomized cohorts within these studies thus combined prior to calculation of pooled effect size across studies. Therefore, RCT and cohort studies were not separated throughout this meta-analysis. Additional information on RCT risk of bias can be found in Fig. 2.

**Fig. 3:**
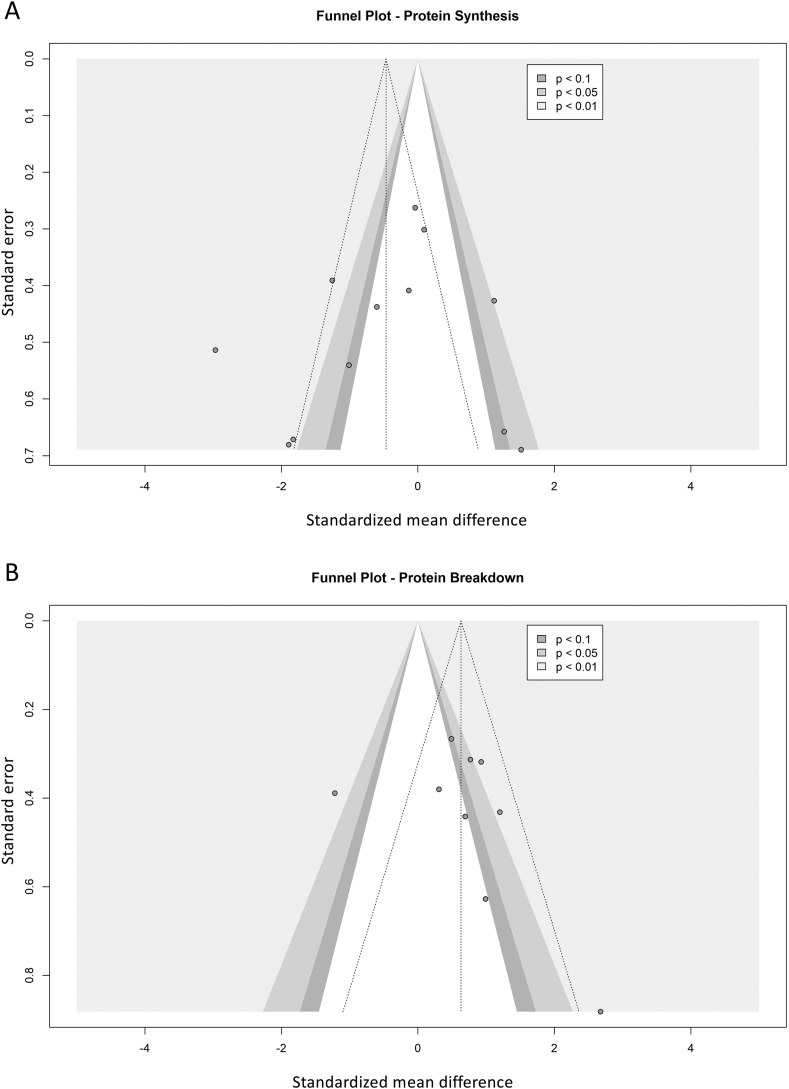
Contour-enhanced funnel plots of protein synthesis (A) and protein breakdown (B) study effects, with significance represented by contour shading at thresholds of p < 0.1, p < 0.05 and p < 0.01.

Publication bias was analyzed via funnel plot and Pustejovky's and Rodger's modified test of linear regression [37], for both measures of protein synthesis and protein breakdown across studies (Fig. 3a and b). Neither tests of publication bias for protein synthesis nor protein breakdown were deemed statistically significant (p = 0.97 and p = 0.57 respectively), although interpretation of these results was limited by the low study numbers included. Several studies in each funnel plot were in range of statistical significance, however due to the high heterogeneity expected across studies due to variation in perioperative care and tracer methodology, all studies were subsequently taken forward for further analyses.

### 3.2 Demographics

Indication for surgery was predominantly colorectal cancer [6,11,12,31,41,42] with the remaining indications a mixture of malignant and benign pathologies [[7], [8], [9],[43], [44], [45], [46], [47]]. Two studies [7,45] included patients having open surgery, with the remaining studies not providing this information [6,8,9,11,12,31,[41], [42], [43], [44],^46^,^47^]. A mix of anesthetic protocols were employed, with five studies [6,41,43,44,47] selectively providing epidural block as part of the anesthetic regimen (either randomized to patients as part of the study design or based on patient need). Five studies did not provide information on anesthetic protocol [9,11,12,31,46], with anesthetic protocol being unknown for one study [45] due to its inclusion based on abstract only. There was varied perioperative nutrition provided to patients across the study period (Table 1), with eight studies [[7], [8], [9],^41^,[43], [44], [45], [46]] including patient cohorts that underwent a preoperative fast (of approximately 12 h or more overnight) or bowel preparation. Tracer methodology utilized within studies came under three categories; those that assessed the direct incorporation of stable isotopes into skeletal muscle that measure fractional synthetic rate (FSR), those that assessed whole-body protein kinetics in the blood via arterio-venous (AV) measures and those that assessed the whole-body kinetics of stable isotope labelling in excreted total or specific urinary substrates (EP). There were five studies that measured protein synthesis via FSR [[6], [7], [8],^44^,^45^], four [9,[41], [42], [43]] via AV, and three [11,12,46] via EP. Studies that utilized direct-incorporation methodology assessed muscle FSR distant from the site of trauma (quadriceps). Five studies [9,[41], [42], [43],^47^] measured protein breakdown via AV, and four [11,12,31,46] via EP. Postoperative timepoints for measures of protein turnover were predominantly between 24 and 72 h, with only one study's measures [42] being performed later than this range at 144 h.

**Table 1:** Patient demographics of studies included.

Article	Eligible Patient Cohort	Number of Patients (Total)	Surgical Procedure	Anaesthesia and Analgesia	Perioperative Nutrition	Stable Isotope Tracer	Sampling Timepoints Included (Pre-: Post-operative)
Tashiro et al., 1991 [11]	Gastric/colorectal surgery	11	Total gastrectomy: 7, hemicolectomy: 3, low anterior resection: 1	Unknown	TPN exclusively, 1.5 g protein/kg/day and 35 kcal/kg/day	[15 N] Glycine; EP	Pre: Not specified Post: 72 h
Lattermann et al., 2002 [41]	General anaesthesia with epidural block/General anaesthesia only	8/8 (16)	Hemicolectomy/colectomy: 2/5, sigmoid resection: 3/1, anterior resection: 3/1, Ileocolic resection: 0/1	General anaesthesia with patients randomised to either epidural or IV morphine postoperatively	∼36 h preoperative fast	L-[1–13C] Leucine; AV	Pre: 0 h Post: 2 h
Carli et al., 1997 [42]	Parenteral nutrition control group	6	All surgery for non-metastatic adenocarcinoma of the rectosigmoid colon	General anaesthesia with postoperative subcutaneous infusion of papaveretum (3–5 mg/h) for 3–4 days	0.1 g nitrogen/kg/day and 20 kcal/kg/day. Nonprotein calories were 60% lipid and 40% carbohydrate. Oral intake was started 6 days before surgery under dietetic supervision, and was then changed to parenteral nutrition at 500 ml Vamin 14, 1 L Intralipid 10% and 1 L dextrose 10% 2 days before surgery and continued for 6 days afterward.	L-[1–13C] Leucine; AV	Pre: 0 h Post: 144 h
Carli and Halliday 1997 [6]	General anaesthesia with epidural block/general anaesthesia only	6/6 (12)	Paramedian incision for non-metastatic adenocarcinoma of the rectosigmoid colon	General anaesthesia with patients randomised to either; epidural maintained for 48 h postoperatively supplemented with papaveretum (8–10 mg) given i.m. Every 8 h or continuous subcutaneous infusion of papaveretum set at 3–8 mg/h	0.1 g nitrogen/kg/day and 20 kcal/kg/day. Nonprotein calories were 60% fat and 40% carbohydrate. Oral intake commenced 6 days before surgery under dietic supervision and changed to parenteral nutrition (500 ml Vamin 14, 1 L Intralipid 10%, 1 L dextrose 10%) 2 days before surgery. Discontinued at midnight day before surgery, recommenced at 4 h postoperatively and maintained for 2 days after surgery.	L-[1–13C] Leucine; FSR	Pre: 0 h Post: 48 h
Carli et al., 2011 [43]	Oral Glucose Nutrition/Oral Whey Nutrition	6/7 (13)	Hemicolectomy/colectomy: 4/4, Sigmoid resection: 0/2, Anterior resection: 2/1	General anaesthesia with epidural or intraoperative IV analgesia; postoperative epidural for 2 days or PCA with opioids	Preoperative fast of ∼24–36 h. Postoperatively, patients were allowed to drink clear fluids unless contraindicated. Clear fluids consisted of a small portion of apple juice (approximately 110 kcal) and Jell-O® (Kraft Foods, Northfield, Illinois) (approximately 70 kcal).	L-[1–13C] Leucine; AV	Pre: −168 h Post: 48 h
Tashiro et al., 1996 [12]	Gastric or colorectal surgery	22	Total gastrectomy, hemicolectomy or lower anterior resection, and lymph node dissection.	Unknown	Parenteral nutrition providing 1.5 g amino acid/kg/day and energy intake of 35 kcal/kg/day. No fat was provided as an energy source. PN was started 7 days prior to the operation and maintained across the study duration. Doses of protein and energy were maintained strictly the same throughout the study.	[15 N] Glycine; EP	Pre: Not specified Post: 72 h
Hammarqvist et al., 2001 [44]	Glutamine PN group	8	Colon resection: 4, rectum resection: 3, retroperitoneal resection: 1	General anaesthesia. 3 patients were also provided with epidural blockade, although this was not provided continuously throughout the study period.	Postoperative parenteral nutrition containing 0.15 g nitrogen/kg/day including an amino acid solution, supplemented with 0.28 g glutamine/kg/day. Energy provided as glucose and fat, calculated as 1.2-fold of caloric need as determined by Harris-Benedict formula. 75% of parenteral nutrition dose administered in first day after operation (25% across following 2 days).	L-[2H5] Phenylalanine; FSR	Pre: 0 h Post: 72 h
Essén et al., 1993 [45]	Saline/Parenteral nutrition	8/9 (17)	Cholecystectomy	Unable to source full-text article.	Saline or parenteral nutrition for 3 days postoperatively.	L-[1–13C] Leucine; FSR	Pre: Unknown Post: 72 h
Tjäder et al., 1996 [7]	Saline	7	Cholecystectomy - subcostal incision	General anaesthesia, with diazepam (5 mg) and pancuronium (0.1 mg/kg) for neuromuscular block, with postoperative IV injections of pethidine (synthetic opioid).	Saline perioperatively 3 ml/kg/h, followed by 35 ml/kg/day postoperatively.	L-[2H5] Phenylalanine; FSR	Pre: 0 h Post: 24 h
López-Hellín et al., 2004 [46]	Fasted/Parenteral nutrition	21/8 (29)	Left hemicolectomy: 9; right hemicolectomy: 5; front rectum resection: 4; Miles' resection: 1; gastrectomy: 1; sigmoidectomy: 1 (21). Left hemicolectomy: 3; Miles' resection: 2; front rectum resection: 1; right hemicolectomy: 1; gastrectomy: 1 (8).	Unknown	Preoperative hypocaloric parenteral nutrition: CHO (28 kJ/kg/day), Amino acids (1 g/kg/day) - followed by either: preoperative fast and postoperative parenteral nutrition of glucose (28 kJ/kg/day) OR TPN (56.1 kJ/kg/day CHO, 56.1 kJ/kg/day Fat, 1.5 g/kg/day Amino acids) administered pre- and post-operatively for 24 h.	[15 N] Glycine; EP	Pre: −72 h Post: 24 h
Essén et al., 1992 [8]	Cholecystectomy patient group	7	Cholecystectomy - subcostal incision	General anaesthesia, with diazepam (5 mg) and pancuronium-bromide (0.1 mg/kg) for neuromuscular block.	Acute fasted study.	L-[1–13C] Leucine; FSR	Pre: 0 h Post: Immediately after surgery
Lattermann et al., 2001 [47]	General anaesthesia/General anaesthesia with epidural block	7/7 (14)	Elective cystoprostatectomy - Ileal neobladder: 6/6, Ileal conduit: 1/1	General anaesthesia/General anaesthesia with epidural block. Epidural terminated immediately after surgery – both patient cohorts received IV Piritramide postoperatively.	Parenteral nutrition from 24 h postoperatively until 10 h before postoperative measurement. 2 g/kg/day xylitol and amino acids, equivalent to 0.15 g of N/kg/day.	[15N2] Urea; AV	Pre: −72 h Post: 72 h
Tashiro et al., 1996b [31]	Gastric or colorectal surgery	22	Total gastrectomy: 11, Hemicolectomy: 4, Low anterior resection: 6, Miles' operation: 1	Unknown	Parenteral nutrition providing 1.5 g of protein and 40 kcal/kg/day, commenced at least 5 days prior to surgery and maintained throughout study period.	[15 N] Glycine; EP	Pre: Not specified Post: 72 h
Carli et al., 1990 [9]	Total abdominal hysterectomy	6	Menorrhagia	Unknown	0.1 g of nitrogen/kg body weight and 1200–1400 calories (5021–5858 kJ)/day was commenced 7 days before surgery by oral intake. The same amount of nitrogen and calories was administered intravenously after surgery starting 4 h from the end of surgery when the cardiorespiratory conditions were stable. The parenteral nutritional support, based on a mixture of glucose, lipid and amino acids (KabiVitrum), was then continued for 4 days after surgery until patients were able to tolerate the pre-operative oral diet again.	L-[1–13C] Leucine: AV	Pre: −48 h Post: 48 h

FSR: fractional synthetic rate; AV: arterio-venous; EP: end-product.

### 3.3 Tracer methodology

#### 3.3.1 Protein synthesis

**Fig. 4:**
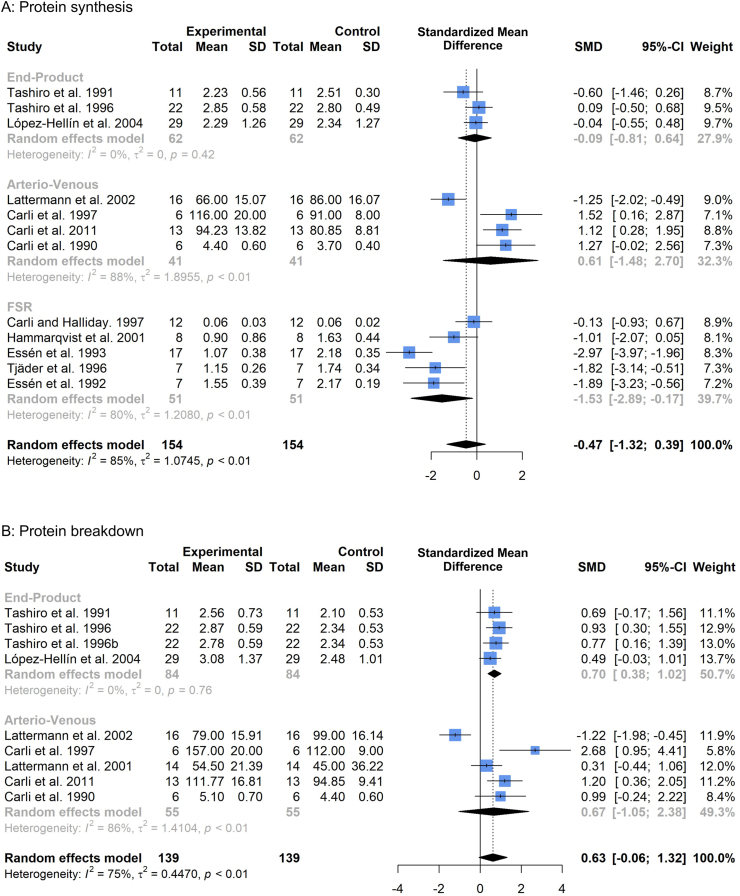
Forest plot illustrating relative changes in protein synthesis (A) and protein breakdown (B), before and after surgery, with studies separated into subgroups by stable isotope tracer methodology. A random-effects, inverse-variance model was used to conduct the meta-analysis.

Subgroup analysis of relative changes in protein synthesis (Fig. 4a) pre-post operation illustrated significant suppressions through direct-incorporation methodology (FSR, SMD -1.53, 95%CI: −2.89 to −0.17, p = 0.03). No significant change was observed in whole-body arterio-venous measures (SMD 0.61, 95%CI: −1.48 to 2.70, p = 0.58) or whole-body end-product measures (SMD -0.09, 95%CI: −0.81 to 0.64, p = 0.82). Overall protein synthesis showed a slight trend for suppression, but this did not reach statistical significance (SMD -0.47, 95%CI: −1.32 to 0.39, p = 0.25).

#### 3.3.2 Protein breakdown

Subgroup analysis of relative changes in protein breakdown (Fig. 4b) before and after surgery demonstrated significant increases via whole-body end-product methodology (SMD 0.70, 95%CI: 0.38 to 1.02, p < 0.001). No significant effect was observed via whole-body arterio-venous measures (SMD 0.67, 95%CI: −1.05 to 2.38, p = 0.45). Overall protein breakdown showed a trend for increase, but this did not reach significance (SMD 0.63, 95%CI: −0.06 to 1.32, p = 0.07).

### 3.4 Preoperative fasting

Nutritional support is a key parameter in the metabolic management of the surgical patient, with recent evidence reinforcing the negative consequences of extended periods of caloric and protein deficits in critically-ill surgical patients [53]. Thus, a key component of many current recommendations on clinical nutrition for the surgical patient advocate the avoidance of prolonged periods of preoperative fasting among elective procedures, particularly within gastrointestinal surgery where bowel preparation has traditionally been common practice [1,19].

#### 3.4.1 Protein synthesis

**Fig. 5:**
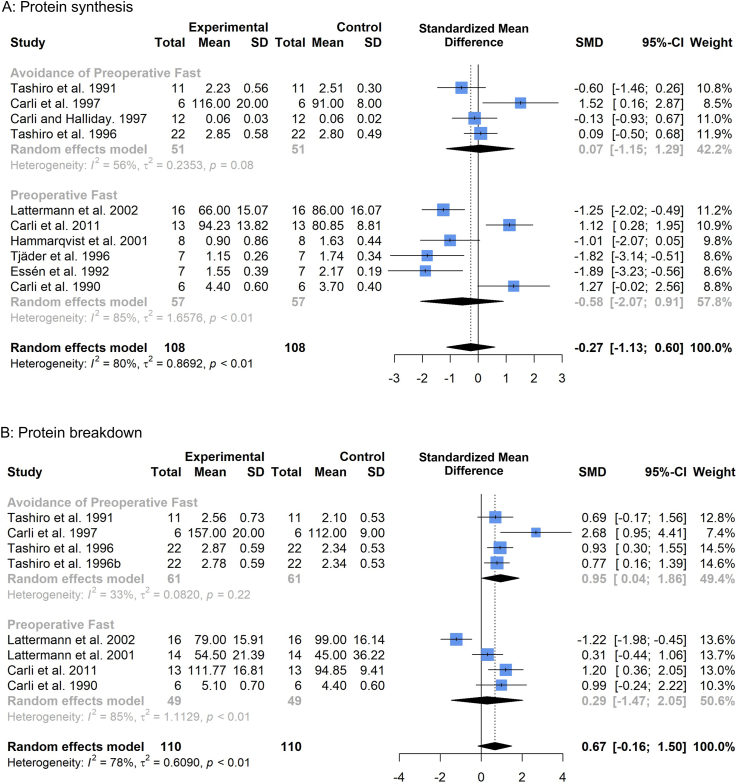
Forest plot illustrating relative changes in protein synthesis (A) and protein breakdown (B), before and after surgery, with studies separated by whether patients underwent or avoided preoperative fast. A random-effects, inverse-variance model was used to conduct the meta-analysis.

In six studies [[7], [8], [9],^41^,^43^,^44^] measuring protein synthesis within this meta-analysis, patients underwent a preoperative fast as part of conventional perioperative care or bowel preparation. In four studies [6,11,12,42], patients did not undergo a preoperative fast (and were receiving consistent nutritional support prior to operation). One study [46] had to be excluded from subgroup analysis of preoperative fasting due to preoperative study measures of protein synthesis being pooled across two patient cohorts; one of which underwent preoperative fasting and the other avoided preoperative fasting. The study author was unable to provide the necessary information to enable inclusion of these cohorts. Incidence of preoperative fast could not be sourced from a further study [45], due to its inclusion on abstract only, and was consequently excluded from the subgroup analysis. Preoperative fast resulted in no significant changes in protein synthesis (SMD -0.58, 95%CI: −2.07, 0.91, p = 0.45, Fig. 5a), although there was high heterogeneity present among studies (*I*^2^: 85%, p < 0.01). Avoidance of preoperative fasting also demonstrated no significant changes (SMD 0.07, 95%CI: −1.15, 1.29, p = 0.92).

#### 3.4.2 Protein breakdown

In four studies [9,41,43,47] measuring protein breakdown patients underwent a preoperative fast, with four studies [11,12,31,42] containing patient cohorts that avoided preoperative fasting or bowel preparation. As before, one study [46] was excluded due to pooled preoperative baseline measures between fasted and non-fasted patients. Avoidance of preoperative fasting resulted in significant increases in protein breakdown (SMD 0.95, 95%CI: 0.04 to 1.86; p = 0.04, Fig. 5b), with fasted patients demonstrating no significant change (SMD 0.29, 95%CI: −1.47 to 2.05, p = 0.76).

### 3.5 Preoperative nutritional management

To further examine the role of preoperative nutrition in the metabolic management of the surgical patient, we examined changes in protein turnover following surgery in patients that received controlled nutritional support opposed to those that didn't, as well as for those articles where this information was unknown.

#### 3.5.1 Protein synthesis

**Fig. 6:**
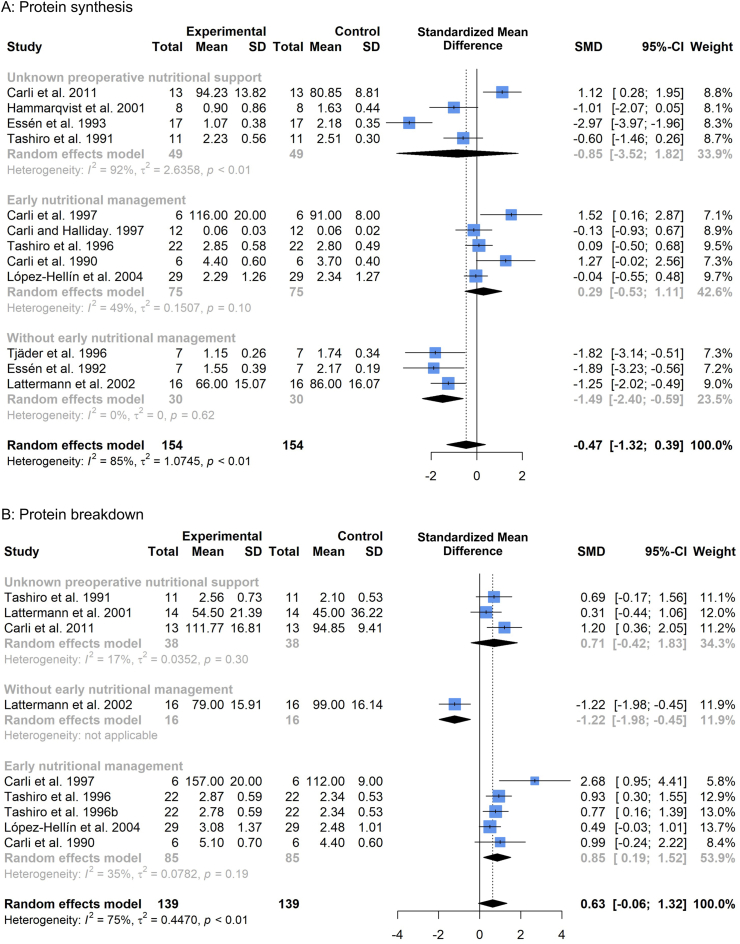
Forest plot illustrating relative changes in protein synthesis (A) and protein breakdown (B), before and after surgery, with studies separated by whether patients received early nutritional management. A random-effects, inverse-variance model was used to conduct the meta-analysis.

Five studies [6,9,12,42,46] provided early nutritional management in the form of controlled dietary intake (Fig. 6a) commenced 3–7 days before surgery. Three studies [7,8,41] did not provide early nutritional management to patients, with this information being unknown for the remaining four studies [11,[43], [44], [45]]. Lack of early nutritional management resulted in significant declines in protein synthesis rates postoperatively (SMD -1.49, 95%CI: −2.40, −0.59, p = 0.001). Postoperative declines in protein synthesis were not present among studies where patients received early nutritional management (SMD 0.29, 95%CI: −0.53, 1.11, p = 0.50). For studies where preoperative nutritional support information was not available, there was a non-significant effect (SMD -0.85, 95%CI: −3.52, 1.82, p = 0.54) and high heterogeneity (*I*^*2*^ = 92%).

#### 3.5.2 Protein breakdown

Five studies [9,12,31,42,46] provided early nutritional management through controlled dietary intake (Fig. 6b) commenced 3–7 days before surgery. Only one study [41] could be confirmed to have not provided preoperative nutritional management, with information on preoperative nutrition unknown for three studies [11,43,47]. Early nutritional management resulted in elevations in (whole-body) protein breakdown (SMD 0.85, 95%CI: 0.19, 1.52, p = 0.01). The study without preoperative nutritional management [41] demonstrated significant declines in (whole-body) protein breakdown (SMD -1.22, 95%CI: −1.98, −0.45, p = 0.002). Studies where information on preoperative nutritional support was unavailable demonstrated a non-significant effect (SMD 0.71, 95%CI: −0.42, 1.83, p = 0.22).

Further subgroup analyses of nutritional support parameters, such as: nutrient composition, preoperative carbohydrate loading and early postoperative resumption of oral feeding, were not possible with the low study numbers contained within this meta-analysis.

### 3.6 Time

**Fig. 7:**
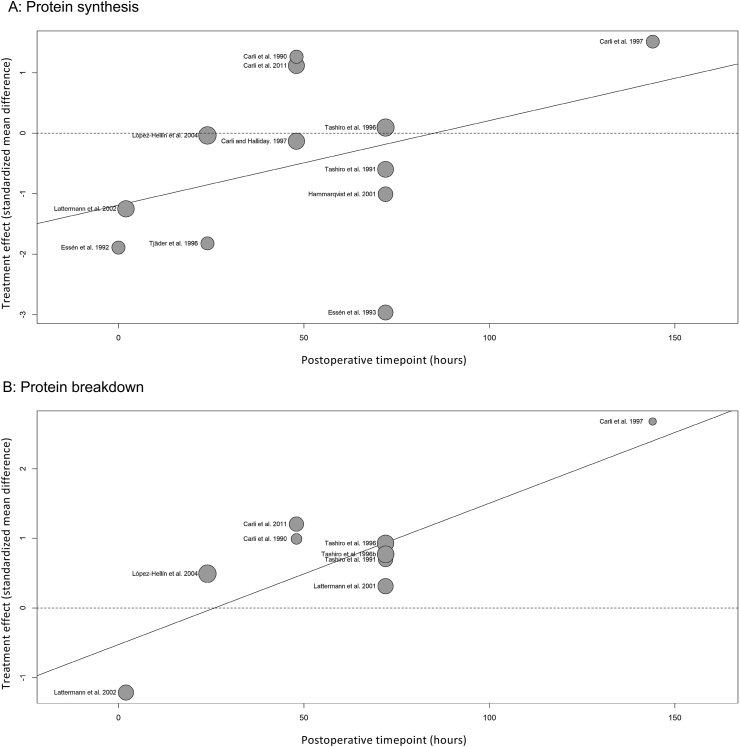
Bubble plot illustrating meta-regression analysis of postoperative changes in protein synthesis (A) and protein breakdown (B), relative to the timepoint (in hours) of postoperative sampling.

Meta-regression of postoperative timepoint sampling (representing the proximity of protein turnover measures to surgery) illustrated a trend for early suppressions in protein metabolism with gradual restoration over time towards baseline values. Protein synthesis demonstrated a non-significant trend (p = 0.21, Fig. 7a), while protein breakdown demonstrated a significant trend (p = 0.01, Fig. 7b). However, interpretation of these findings is limited by the small study numbers and with respect to protein breakdown measures, potentially impacted by study homogeneity stemming from three data sets by the same author [11,12,45] being grouped closely together within the meta-regression analysis (Fig. 7b).

### 3.7 Anesthesia, epidural blockade and severity of surgical trauma

There was insufficient reporting of open *vs*. laparoscopic procedures to enable comparisons between the extent of surgical trauma and measures of protein synthesis/breakdown, with specification of these parameters contained within only two studies [7,45]. Anesthetic regimens differed but there were insufficient study numbers to group by minor modalities (specific drug regimens to induce general anesthesia, Table 1). Only three studies [6,41,47] included a patient cohort where all participants received epidural blockade as part of their anesthetic treatment, with a further two studies [43,44] containing patient cohorts receiving mixed anesthetic treatment with and without epidural administration and five studies [9,11,12,31,46] not providing this information. Therefore, no subgroup analyses were performed on these parameters within this meta-analysis.

## 4 Discussion

### 4.1 What our study found

Assessment via stable isotope techniques demonstrated trends for reductions in protein synthesis and elevations in protein breakdown to occur following abdominal surgery, within the context of varied perioperative care. These were characterized by significant suppressions in skeletal muscle protein synthesis that were not reflected within whole-body measures and significant increases in whole-body end-product but not arterio-venous protein breakdown.

The findings of this meta-analysis suggest that suppressions in postoperative protein synthesis were not contributed by preoperative fasting but are more importantly regulated by whether sufficient caloric and protein intake of patients was met in the days leading up to their operation. Avoidance of preoperative fasting resulted in elevated protein breakdown that was not reflected in patients that underwent preoperative fast, with early nutritional management also resulting in elevated protein breakdown postoperatively and lack of early preoperative diet management resulting in suppressed protein breakdown. Care must be taken in the interpretation of these findings, as only whole-body protein breakdown was measured and based on the findings of this meta-analysis, these measures likely do not accurately reflect the protein kinetics of skeletal muscle. However, it overall appears that sufficient preoperative caloric and protein intake facilitates increased rates of protein turnover postoperatively. Meta-regression provides limited support for postoperative suppressions in protein turnover to be most acute during the immediate postoperative period, and to thereafter increase with time. This may suggest early recommencement of nutritional support to be vital in the immediate postoperative period, although examination of this effect was unfortunately not possible within this meta-analysis.

### 4.2 What is available in the literature

Variation in stable isotope assessment of protein kinetics through techniques measuring distinct metabolic pools has previously been observed in surgical patients undergoing coronary artery bypass grafts [54], who demonstrated significant reductions in muscle protein synthesis (-∼36%) but notable increases in plasma fibrinogen (+∼177%) and albumin (+∼45%) synthesis postoperatively. Discrepancy between these metabolic pools has been suggested to be a result of the different metabolic demands these pools are subject to following surgical trauma [55], wherein amino acids are mobilized from skeletal muscle to necessitate energy and healing demands and liver protein metabolism is accelerated to promote the production of acute phase reactants. Increases in whole-body protein turnover associated with healing-driven hypermetabolism, would be in line with traditional observations correlating early wound healing and elevated urinary nitrogen excretion rates among patients in receipt of good preoperative nutrition [4], where administration of parenteral nutrition during the postoperative period appears to augment hypermetabolism compared to hypocaloric glucose [56], but simultaneously results in improved nitrogen balance [57]. Our findings support these concepts. There is a clear disparity between the postoperative synthetic responses of muscle and whole-body, with muscle alone demonstrating significant reductions postoperatively. Preoperative nutrition aimed at meeting the caloric and protein requirements of patients attenuates reductions in protein synthesis and elevates protein breakdown, with lack of unified magnitude in these responses likely reflective of the inclusion of direct-incorporation methodology within studies measuring protein synthesis. This reaffirms the importance of applying stable isotope techniques specific to the metabolic pool of interest to accurately study protein metabolism.

### 4.3 Strengths and limitations

Only studies utilizing stable isotope tracer methodologies were included in this meta-analysis, with these believed to provide the most comprehensive insight into protein kinetics within the surgical patient [58]. This meta-analysis is strengthened by a pre-test post-test design that enables the accurate determination of relative changes in protein turnover for each patient cohort through measurement of protein turnover in a controlled nutritional state before and after surgery (either postabsorptive or postprandial stable isotope measures). Many previous insights into perioperative catabolism and the investigation of care strategies aimed at modulating the catabolic response to surgery (as measured through stable isotope techniques) have utilized RCT designs centered on postoperative comparisons between cohorts, with many of these studies measuring postabsorptive protein turnover at baseline but postprandial protein turnover postoperatively [[59], [60], [61], [62], [63], [64], [65]] (Supplementary Table 2). Although this design is suitable in discerning the benefits of care strategies aimed at ameliorating catabolism through between-patient comparisons, they are limited in their ability to discern the mechanistic drivers of these changes during the surgical care period within patients.

However, this exclusion resulted in low study numbers that was unfortunately further contributed by the omission of several eligible articles [10,[48], [49], [50], [51]] (Supplementary Table 2) that did not present the continuous data necessary for inclusion in this meta-analysis. Additionally, data from several included papers had to have their means and standard deviations estimated from median and interquartile range [44,45,47]; although this was performed using an established method [35] that has been employed in numerous published meta-analyses. The use of only continuous data to calculate pooled effect sizes does, however, aid in further strengthening the validity of results in the context of a highly heterogenous data set. With reference to the *I*^2^ statistic; for both protein synthesis and protein breakdown, only whole-body [EP] measures under tracer subgrouping had an *I*^2^ statistic <25%, with the majority of subgroups having an *I*^2^ statistic >50%. With low study numbers, it is difficult to discern whether this reduced heterogeneity may be due to the necessary control of nutritional intake to enable accurate stable isotope measures [66] or whether it is influenced by many of these studies being performed by the same research group potentially utilizing standardized procedures [11,12,45]. Overall, heterogeneity for protein synthesis was 85% and 75% for protein breakdown, potentially lower due to the lack of direct-incorporation measures. This high variation must be taken into consideration when evaluating the findings of this meta-analysis, but with such low study numbers this observation is not unexpected, even within a strictly defined meta-analysis design.

Subgroup analyses investigating the impacts of preoperative nutrition on postoperative changes in protein synthesis and breakdown demonstrated preoperative fasting to result in high heterogeneity among study results (*I*^2^ = 85% for both protein synthesis and breakdown). Less heterogeneity was present among studies where preoperative fasting was avoided (*I*^2^ = 56% for protein synthesis, *I*^2^ = 33% for protein breakdown). For studies measuring protein synthesis, early nutritional management presented moderate heterogeneity (*I*^2^ = 49%) and lack of early nutritional management presented low heterogeneity (*I*^2^ = 0%), with unknown nutritional management demonstrating expectantly high heterogeneity (*I*^2^ = 92%). Interpretation of heterogeneity regarding nutritional management for studies measuring protein breakdown is limited due to the presence of only one study that did not receive early nutritional management. Overall, heterogeneity was low to moderate for these results. Following the high heterogeneity present among tracer methodology subgroup analyses, mixed tracer subgrouping by nutritional parameters resulted in relatively low heterogeneity. These observations may support preoperative nutrition to exert effects on the postoperative response of protein turnover, likely through the administration of regimented dietary intake providing adequate caloric and protein intake among patients for their metabolic demands. Unfortunately, varied pre- and postoperative nutritional regimens and varied postoperative nutritional administration prevented examination of these parameters with the low study numbers contained within this meta-analysis.

## 5 Conclusions

Elective abdominal surgery elicits suppressions in skeletal muscle protein synthesis remote to the site of trauma that are not reflected on a whole-body level. Lack of uniform changes across whole-body tracer techniques are likely due to contribution from tissues other than skeletal muscle and complicate the discernment of mechanistic processes driving postoperative skeletal muscle wasting. Future work should focus on tissue-specific stable isotope approaches to comprehensively characterize the protein turnover responses of skeletal muscle, within the context of enhanced recovery after surgery care strategies.

## Funding

This work was supported by the 10.13039/501100000265Medical Research Council [grant number MR/K00414X/1]; 10.13039/501100000341Arthritis Research UK [grant number 19891]. Matthew Jaconelli is in receipt of a funded PhD studentship from the Medical Research Council/Versus Arthritis Centre for Musculoskeletal Ageing Research. Matthew S. Brook is a recipient of a Research Fellowship from the European Society for Clinical Nutrition and Metabolism (ESPEN).

## Author contributions

Study design – All authors.

Data extraction and analysis – MJ and MSB.

Writing of the manuscript – All authors.

Critical review – PLG, PJA and DNL.

Final approval – All authors.

## Conflicts of interest

None of the authors has a conflict of interest to declare.
