# Supplementary material for: The effects of elective abdominal surgery on protein turnover: A meta-analysis of stable isotope techniques to investigate postoperative catabolism
Source: Clin Nutr. 2022 Mar;41(3):709–22. doi: 10.1016/j.clnu.2022.01.023 (PMC8902515; doi:10.1016/j.clnu.2022.01.023)
Supplement: Multimedia component 1 [file mmc1.docx]

**Supplementary Table 1:** PICO statement

| Patients | Elective abdominal surgical patients |
| --- | --- |
| Intervention | Abdominal operation |
| Comparison | Pre-postoperative comparison within patient cohorts |
| Outcomes | Protein turnover measured via stable isotope tracers |

**Supplementary Table 2:** Relevant studies excluded

| **Article** | **Reason for Exclusion** |
| --- | --- |
| Lattermann et al. 2007 [59] | Preoperative protein turnover assessed under post-absorptive feeding conditions; postoperative protein turnover assessed under post-prandial feeding conditions |
| Schricker et al. 2008 [60] | Preoperative protein turnover assessed under post-absorptive feeding conditions; postoperative protein turnover assessed under post-prandial feeding conditions |
| Lugli et al. 2010 [61] | Preoperative protein turnover assessed under post-absorptive feeding conditions; postoperative protein turnover assessed under post-prandial feeding conditions |
| Lattermann et al. 2003 [62] | Preoperative protein turnover assessed under post-absorptive feeding conditions; postoperative protein turnover assessed under post-prandial feeding conditions |
| Schricker et al. 2004 [63] | Preoperative protein turnover assessed under post-absorptive feeding conditions; postoperative protein turnover assessed under post-prandial feeding conditions |
| Schricker et al. 2005 [64] | Preoperative protein turnover assessed under post-absorptive feeding conditions; postoperative protein turnover assessed under post-prandial feeding conditions |
| Schricker et al. 2013 [65] | Preoperative protein turnover assessed under post-absorptive feeding conditions; postoperative protein turnover assessed under post-prandial feeding conditions |
| Tjäder et al. 2004 [33] | Heterogenous surgical population including patients undergoing vascular procedures. |
| Carli and Halliday. 1996 [10] | Unable to source necessary data from article or author(s) |
| Carli et al. 1991 [48] | Unable to source necessary data from article or author(s) |
| Carli et al. 1991b [49] | Unable to source necessary data from article or author(s) |
| Carli et al. 1997 [50] | Unable to source necessary data from article or author(s) |
| López Hellín et al. 2008 [51] | Unable to source necessary data from article or author(s) |
| Carli et al. 1990 [52] | Unable to source necessary data from article or author(s) |
